# Val50Met hereditary transthyretin amyloidosis: not just a medical problem, but a psychosocial burden

**DOI:** 10.1186/s13023-021-01910-5

**Published:** 2021-06-10

**Authors:** Juan González-Moreno, Aina Gaya-Barroso, Inés Losada-López, Adrián Rodríguez, Teresa Bosch-Rovira, Tomás Ripoll-Vera, Mercedes Usón, Antoni Figuerola, Cristina Descals, Carles Montalà, María Asunción Ferrer-Nadal, Eugenia Cisneros-Barroso

**Affiliations:** 1 Servicio de Medicina Interna, Hospital Universitario Son Llàtzer, Crta Manacor Km 4, 07198 Palma, Balearic Islands Spain; 2 Balearic Research Group in Genetic Cardiopathies, Sudden Death and TTR Amyloidosis, Instituto de Investigación Sanitaria de las Islas Baleares (IdISBa), Palma, Balearic Islands Spain; 3 Servicio de Medicina Interna, Hospital Universitario Son Espases, Carretera de Valldemossa, 79, 07120 Palma, Balearic Islands Spain; 4 Servicio de Cardiología, Hospital Universitario Son Llàtzer, Crta Manacor Km 4, 07198 Palma, Balearic Islands Spain; 5 Servicio de Neurología, Hospital Universitario Son Llàtzer, Crta Manacor Km 4, 07198 Palma, Balearic Islands Spain; 6 Servicio de Nefrología, Hospital Universitario Son Llàtzer, Crta Manacor Km 4, 07198 Palma, Balearic Islands Spain

**Keywords:** Hereditary transthyretin amyloidosis, Burden of disease, Rare disease, Well-being, Caregivers

## Abstract

**Background:**

Hereditary transthyretin (TTR) amyloidosis (ATTRv) is a heterogeneous disease with a clinical presentation that varies according to geographical area and TTR mutation. The symptoms of Val50Met-ATTRv are mainly neuropathic and progress to complete disability and death in most untreated patients within 10 to 15 years of diagnosis. The neurological effects may also be accompanied by gastrointestinal impairment, cardiomyopathy, nephropathy and/or ocular deposition. The disease is thus associated with a high degree of patient disability. Accordingly, we aimed to describe the psychosocial burden associated with ATTRv in a group of patients, asymptomatic Val50Met carriers, relatives and caregivers in the endemic focus of the disease in Majorca via a survey addressing various aspects related to psychosocial burden. We performed a an observational, descriptive, cross-sectional and multicentre study in order to analyze the prevalence of self-reported impact of ATTRv disease upon their daily life. In addition to the self-knowledge, fear and burden related to the disease. The survey was disseminated during the regular follow up at the outpatient clinic of the Hospital Universitario Son Llàtzer and during the meetings organized by the Andrade’s Disease patients’ advocacy group from the Balearic Islands. These meetings were attended also by subjects followed up by the Hospital Universitario Son Espases and their caregivers and relatives. Survey was self-administrated. No intervention was done by the investigators. 85 subjects completed the survey: 61 carrying the TTR-V50M variant and 24 caregivers or relatives.

**Results:**

Our study revealed that, although most of the population studied had had prior contact with ATTRv through affected relatives, there was still a lack of information regarding disease diagnosis. Fear of the genetic test result and psychological issues were common in our population. Moreover, the disease had a stronger impact on the daily life of our patients than that of our asymptomatic carriers. Autonomic symptoms were the main source of burden for relatives and caregivers.

**Conclusion:**

Our survey results show high psychosocial burden associated with Val50Met-ATTRv in our area.

**Supplementary Information:**

The online version contains supplementary material available at 10.1186/s13023-021-01910-5.

## Introduction

Hereditary transthyretin (TTR) amyloidosis (ATTRv), formerly known as familial amyloidotic polyneuropathy (FAP), is characterised by the deposition of amyloid fibrils derived from the accumulation of unstable conformations of the TTR protein [1]. More than 120 mutations have been described in the *ttr* gene. Of these, Val50Met (V50M) is the most common variant in patients with hereditary transthyretin amyloidosis with polyneuropathy (ATTRv-PN) as the main manifestation [2]. V50M is also the most frequent variant in the two largest foci in Spain: Majorca and Valverde del Camino, where ATTRv is considered endemic [3, 4].

ATTRv presents in many forms and exhibits a considerable variability in signs and symptoms among individuals and across geographic locations [1]. The various organs involved are the nerves, gastrointestinal system, heart, kidneys and eyes. The symptoms of V50M-ATTRv are mainly neuropathic and include peripheral (sensory and motor) and autonomic neuropathy. The neurological disease is frequently associated with gastrointestinal impairment, cardiomyopathy, ocular manifestations related to amyloid deposition in the eye and nephropathy [5].

ATTRv diagnosis at an early stage is essential for a timely treatment to halt disease progression. Early recognition remains a challenge, with misdiagnosis often delaying diagnosis [6–11]. Even today, there is still a significant delay in the diagnosis of ATTRv, especially among individuals without an identified family history for the disease due to incomplete penetrance of the gene, unusual de novo mutations or previously misdiagnosed parents [6–8, 11, 12].

Due to the progressive and sometimes fatal nature of the disease and its inheritance, the disease can have an immediate and strong psychosocial impact on patients’ lives and those of their families from disease diagnosis, even in those informed that they asymptomatically carry the pathogenic mutation [13].

The disease manifests in adulthood and is chronic, with a progressive development that can continue for more than a decade. Therefore, the onset of the disease can significantly affect the lives of both patients and their relatives, both socioeconomically and in terms of the family dynamics, as has already been described in the case of other chronic diseases [14].

Accordingly, the aim of this study was to describe the psychosocial burden associated with ATTRv in terms of personal, social and familial aspects in a group of patients, V50M carriers, relatives and caregivers in the endemic focus of the disease in Majorca.

## Methods

### Study design and approval

This was an observational, descriptive, cross-sectional and multicentre study. In no case did this study interfere with the researcher’s decision concerning the most appropriate medical care or treatment for a patient. The study was approved by the ethics committee (approval number: IB3463/17) of the Balearic Islands before patient recruitment started.

### Study population and sample

Eligible participants were patients aged 18 years or older with a documented pathogenic mutation in the TTR gene and family caregivers who accompanied them to the regular ATTRv consultation at the internal medicine service of University Son Llàtzer Hospital or to meetings organised by a patient advocacy group, attended also by patients of University Son Espases Hospital. Individuals were excluded if they were unable to sign the informed consent document. V50M-ATTRv patients and relatives who agreed to participate in the study were recruited from January 2019 until December 2019.

### Survey

The survey participants answered a questionnaire with a maximum of 51 questions, which were prepared according to the questionnaires previously published for ATTRv [15]. This survey included questions on demographic characteristics, disease duration and stage related to ambulatory ability, mutation type, family history, self-perception of anxiety or depression and influence of the disease on daily life and income.

For the caregivers, the questionnaire assessed items such as time spent taking care of an ATTRv patient, changes to working life due to the impact of patient care and the impact of the disease on household finances.

Likewise, the patients’ mutation, current age, sex, marital status, number of children and professional situation were recorded, as well as if the patients considered themselves a carrier or symptomatic. If symptomatic, the stage, age at disease onset, age at diagnosis (if applicable) and maternal or paternal inheritance were also recorded.

The survey was presented by a single investigator and, after an informed consent signature was provided, was completed by the participant without any help.

### Statistical analysis

A descriptive study was performed to investigate the impact of the disease on the personal, social and familial variables included in the study. All categorical variables are expressed as numbers and percentages. Not all questions were answered by all of the participants. The reported percentages were thus calculated based on the number of respondents who answered each item. In addition, analyses of the differences between the different populations defined by the secondary variables collected, such as disease stage, age and sex, were performed using the Fisher exact test.

## Results

### Survey completion

Overall, 85 people completed the survey. Of them, 34 (40.0%) were symptomatic patients, 22 (64.7%) with stage I of the disease and 12 (35.3%) with stage II or higher. The survey was also completed by 27 asymptomatic carriers (31.8%) and 24 relatives/caregivers (28.2%) (Tables 1, 2).

**Table 1:** Descriptive characteristics of the sample

	Asymptomatic carriers (N = 27)	ATTRv patients (N = 34)
Sex (female)	16 (59.2)	15 (44.1)
Age (> 50 years)	11 (40.7)	23 (67.6)
Marital status		
Married or unmarried partner	13 (48.1)	22 (64.7)
Divorced	1 (3.7)	2 (5.8)
Widowed	3 (11.1)	3 (8.8)
Single	10 (37.0)	7 (20.6)
With children	15 (55.5)	24 (70.6)
Education level		
No formal education	0 (0)	1 (2.9)
Primary or secondary school level	14 (51.8)	19 (55.8)
Job training	6 (22.2)	8 (23.5)
Bachelor's degree or higher	7 (25.9)	6 (17.6)
Working situation		
Active	16 (59.3)	6 (17.6)
Retired	6 (22.2)	19 (55.8)
Sick leave	1 (3.7)	3 (8.8)
Unemployed	0 (0)	3 (8.8)
Student	4 (14.8)	2 (5.9)

Results are expressed as number and percentage

**Table 2:** Demographic characteristics of the relatives/caregivers (N = 24)

	N (%)
Sex (female)	14 (58.3)
Age (> 50 years)	14 (58.3)
Marital status (n = 24)	
Married or unmarried partner	22 (91.6)
Divorced	1 (4.5)
Single	1 (4.5)
With children	19 (79.2)
Education level (n = 22)	
Primary or secondary school level	13 (59.1)
Job training	6 (27.3)
Bachelor's degree or higher	3 (13.6)
Working situation (n = 21)	
Active	10 (47.6)
Retired	7 (33.3)
Unemployed	3 (14.3)
Student	1 (4.8)
Caregiver's illness (n = 22)	
Yes	5 (22.7)
No	17 (77.3)

Results are expressed as number and percentage

### V50M-ATTRv patients and asymptomatic carriers

The main demographic characteristics of the V50M population (n = 61) are summarised in Table 1.

### Family history

Most of the participants (43 of 57; 75.4%) had some experience of the disease before receiving their own diagnosis, mainly through an affected parent (21 of 57; 36.8%) or another family member (18 of 57; 31.6%). The disease had manifested in 34 of the 61 parents of the participants surveyed (55.7%). The affected parent was the mother for 14 of the 34 participants (41.2%) and the father for 20 (58.8%). Most participants were older than 25 years of age at the onset of their parent’s disease (21 of 34; 61.8%). In addition, 16 of the 34 sick parents (47.0%) were deceased. Most of the participants who lost a parent with ATTRv (12 of 16; 75%) were older than 25 years of age when their parents died. Most participants did not take care of their parents (35 of 54; 64.8%), probably because their parents did not need care (only 3 of the 35 [8.6%] parents of these participants who were not caregivers for their parents died due to the disease). Moreover, most did not need to employ a caregiver (94.4%).

### Knowledge of their own disease

Diagnosis was made between 5 and 10 years before the survey in half of the patients included in the study. Although most of the participants had family affected by the disease, only 8.2% (5 of 61) of the total sample marked their status and identified themselves as a relative of other patients. Among the 27 asymptomatic carriers, 14 (51.8%) assumed that they had polyneuropathy. Additionally, 20 of these participants (74.1%) responded to the question related to the time elapsed since disease diagnosis.

### Diagnosis

Most participants (32 of 50; 64%) indicated that they had access to genetic counselling, largely motivated by family history (23 of 32; 71.9%) or by their physician (7 of 32; 21.8%). Only 2 of the 32 participants who received genetic counselling (6.3%) directly requested it. The remaining 18 of the 50 participants (36%) who answered that they did not have access to genetic counselling accessed the genetic test directly through a regular medical consultation. Fear of knowing the result of the genetic test was felt by 26 of 53 participants (49.1%). Most of the participants (49 of 53; 92.4%) received the genetic test result less than 6 months after the sample collection.

### Impact of the disease

When asked about whether and how the disease had affected their lives, 19 of 56 participants (33.9%) answered that the condition had affected study or work plans. In 13 of 54 surveyed participants (24.1%), the disease had impacted their family plans while 9 of 55 participants (16.4%) felt that the disease had altered their friendships. The disease had had an economic impact on 17 of the 57 participants (29.8%) who completed the survey. The disease had a greater impact on all of the areas assessed in patients than in asymptomatic carriers (*p* < 0.05). Only 8 of 54 surveyed participants (14.8%) received state aid, although none described what type of aid they received.

For those whose family plans were altered by the disease (n = 13), 6 (46.1%) were able to have children through preimplantation genetic diagnosis (PGD) and 4 (30.8%) decided not to have more children after disease diagnosis.

Although almost half of the study population belonged to a patients’ association, this was not the main resource for information on the disease. More than one-third of the survey population used more than one information resource; 40 of 58 participants (69.0%) identified their physician as their primary information resource while 22 (37.9%) cited their own relatives.

About 15 of the 57 participants (26.3%) reported psychological or psychiatric problems related to the diagnosis of the disease or the identification of the disease mutation. Women were more likely to have such problems (*p* < 0.05). No statistically significant differences in the frequency of these problems were found between asymptomatic carriers and diagnosed patients. The most common problems included depression and anxiety and started at diagnosis in 50% of the participants and at symptom onset in 31.2%. In addition, 60% of the participants who reported psychological or psychiatric problems sought professional help from a psychologist or psychiatrist and 50% required treatment with anti-depressants and/or tranquilisers.

Finally, participants were asked about the degree of satisfaction with the disease-related care. In total, 94.5% of the participants believed that the care that they received at the time of diagnosis was remarkable or excellent and 96.2% had the same opinion regarding the care received during disease follow-up.

### Relatives/caregivers

Some of the patients attended their regular consultation with their relatives or caregivers. In total, 24 relatives agreed to take the survey: 15 were the patient’s partner (62.5%), 4 were the patient’s offspring (16.7%), 4 were the patient’s parents (16.7%) and 1 individual did not answer to this question. The disease was diagnosed less than 2 years before the survey in 22.7% of the relatives (5 of 22), between 2 and 5 years in 36.3% (8 of 22), between 5 and 10 years in 9.1% (2 of 22) and more than 10 years in 31.8% (7 of 22). Most of the accompanied patients (15 of 21; 71.4%) were asymptomatic carriers or stage I patients. In addition, 80.9% of the companions (17 of 21) spent less than 5 h per day caring for their relatives. Finally, 77.3% of the caregivers (17 of 22) were healthy; the most common diseases were diabetes and asthma. The demographic characteristics of the companions are summarised in Table 2.

Family caregivers were asked how they perceive the impact of patients’ symptoms in their daily life. For this purpose, family caregivers were asked to punctuate how much they are affected by the most common symptoms suffered by patients. Family caregivers answered that erectile dysfunction (6.8 ± 4.2/10—scale of 1 to 10 to indicate how much it has been affected), fatigue, dizziness and loss of balance (5.3 ± 3.2/10) were the symptoms suffered by patients with the strongest impact on their daily life. Family caregivers were also asked about their perception about the effects of different treatments to control patients’ symptomatology. They indicated that treatment to control blood pressure (7.4 ± 1.1/10) and cardiac function (7.1 ± 1.4/10) had positive effects on patients’ symptoms. However, in family caregivers opinion, treatments only partly helped to control fatigue (6.0 ± 1.4/10) and insomnia (6.0 ± 1.4/10) and failed to control neuropathic pain (4.6 ± 2.1/10) and digestive symptomatology (4.2 ± 3.0/10).

Family caregivers generally did not have health issues and ATTRv disease had barely affected their study or work plans, their family and friendships or their incomes. Most (15 of 17, 88.2%) did not receive any state aid.

## Discussion

From this psychosocial survey completed by ATTRv patients, asymptomatic TTR mutation carriers, relatives and caregivers, we obtained three main findings: (a) some disease concepts (i.e., the difference between mutation carriers and patients) are not clearly understood by our population; (b) the psychosocial burden begins once individuals learn about the presence of a mutation; and (c) autonomic symptoms are the main source of caregiver burden.

Participants were asked about their self-perception of the disease and, although they had already had contact with this rare disease, there was confusion regarding the conditions of patients and carriers because almost half of the asymptomatic carriers believed that they also have polyneuropathy. This could be expected because ATTR has variable penetration and a highly variable disease onset [16–18]. To avoid the stress produced by the lack of information, health education programmes should be introduced and programmes should be created by joint forces between clinicians and patient advocacy groups.

For 88.5% of the participants, diagnosis of the disease or identification of the disease-related mutation was considered a life-changing experience that altered their study or work plans and family plans. The impact of the knowledge of the TTR mutation was particularly notable in asymptomatic carriers. Because ATTRv is a hereditary disease and endemic in our area, it was expected that most asymptomatic carriers would have closely experienced the clinical impact of the disease. Fear and uncertainty are common at disease diagnosis and mutation identification and counselling and clear information are essential to reduce stress. There is increasing evidence that chronic disease care can be improved by stimulating the mental well-being of patients [19]. Thus, introduction of a psychosomatic approach to the multidisciplinary care of this rare disease may help to reduce the psychosocial burden caused by ATTRv in patients, relatives and caregivers.

As frequently reported by patients, autonomic symptoms such as dizziness and orthostatic hypotension represent the highest burden for caregivers. Moreover, most of the symptoms were reported as not being improved by treatment. V50M-ATTRv can present, particularly in early-onset disease, with mainly autonomic dysfunction. Some manifestations, such as erectile dysfunction and digestive problems, can severely diminish the quality of life of patients, especially the younger population. This indicates the importance of including autonomic symptoms and quality of life questionnaires in treatment objectives in clinical practice and clinical trials, as already proposed by others [20–22]. Moreover, the impairment in daily life activities and loss of ambulation abilities worsen the perception of quality of life. Supervised and home-based exercise training programmes have been already proven to improve and preserve walking capacity and normal activity in liver patients transplanted due to ATTRv [23]. Consequently, the introduction of specific training programmes designed and managed by psychotherapists should be considered when the multidisciplinary approach to the care of ATTRv patients is being developed.

Among those patients who reported an impact of the disease on their work or study plans, most of them indicated that they had to stop working due to pain, stress or disability. Some had to adapt their schedule because they were unable to handle work-related stress or the fatigue related to the disease. Diseases that impact daily life, especially those that manifest at early ages, can lower lifetime earnings, savings and wealth accumulation, which can influence living standards in all life stages [24]. The disease was reported to lower the incomes of 29.8% of those surveyed, with its impact on patient income more important for those older than 50 years of age and in advanced stages of the disease, independently of the time since diagnosis.

Most of the patients indicated that the disease did not affect their plans to have a family because they were diagnosed after they had already had children. It was only in those who were diagnosed before they had children or had expanded their family that the disease stopped them from having more children or compelled them to rely on PGD to avoid the transmission of the TTR mutation.

The population studied belongs to the largest endemic focus of the disease in Spain. Most of the participants have relatives already diagnosed with the disease. This population has a high priority for genetic counselling, especially in individuals who are close to the age of onset of their already diagnosed relatives [25]. This is why genetic counselling at our hospital is usually performed directly at the multidisciplinary monographic consultation for ATTRv disease by the physicians involved in the follow-up of this disease, instead of the patients being referred to a genetic consultation [26]. Accordingly, most patients are not aware that they are already undergoing genetic counselling. The incorporation of a genetic counsellor into the multidisciplinary unit should improve informational and emotional issues associated with the genetic test and the hereditary features of this disease.

The sample in this study only included 14.1% of participants in stage II or higher. This means that most of the participants had mild symptoms and preserved ambulatory capability without any help. Thus, most of the participants answered that they did not need to hire a caregiver, probably because they are still in the early stages of the disease. However, we do not know if those who were in later stages and did not hire a caregiver (85.7%) received aid from relatives.

Patients in stage 1 of the disease and asymptomatic carriers mainly attended their medical consultation or meetings organised by the patient advocacy group with at least one relative. Relatives also completed a specific survey to evaluate the impact of the disease on their lives. For most of the relatives, the contact with the disease was recent and, because patients are in the early stages of the disease, the relatives do not invest much time in caring for them because they are self-sufficient.

The survey also wanted to know the opinions of the participants on the medical care received during disease diagnosis and follow-up. Patients rate the care received in both scenarios as remarkable or excellent. The patients included in this study are followed up by a multidisciplinary team, as described by Losada et al. [26]. This multidisciplinary approach aims to cover all of the medical needs of these patients in a timely manner, avoiding delays to diagnosis and any treatment required. Moreover, the multidisciplinary approach is designed to decrease the number of hospital visits required by these patients and to regularly organise health education programmes to provide updates on the disease. The results of this survey indicate that patients value the existence of a medical team dedicated to this rare disease.

## Limitations

The study sample was small because ATTRv is a rare disease. Moreover, all participants recruited in this study come from the ATTRv endemic region of Majorca in the Balearic Islands. Thus, the results obtained in this study have to be carefully taken into account and further studies are needed to confirm the burden caused by the disease in the overall Spanish population and in other countries. Participation was limited to patients who were able to write or type and were able to attend their regular consultation at the hospital or the meetings organised by the patient advocacy group. Accordingly, patients with a major disability are probably underrepresented in this study.

## Conclusions

These results highlight the burden of ATTRv for patients and their relatives. Patients, carriers and their relatives and caregivers face challenges beyond medical issues. The impact of this disease is mainly on quality of life and mental health, not only for patients, but also for asymptomatic carriers and their relatives.

## Supplementary Information

**Additional file 1.** Patient and caregiver surveys.

## Data Availability

The results described here are the analysis of the surveys answered by patients and caregiver without any other intervention. Patient and caregiver surveys are provided in Additional file 1.

## Abbreviations

ATTRv: Herditary transthyretin amyloidosis
ATTRv-PN: Herditary transthyretin amyloidosis with polyneuropathy
FAP: Familial amyloid polyneuropathy
PGD: Preimplantation genetic diagnosis
TTR: Transthyretin
V50Met: Replacement of valine with methionine at position 50
Val50Met: Replacement of valine with methionine at position 50
