# Supplementary material for: Val50Met hereditary transthyretin amyloidosis: not just a medical problem, but a psychosocial burden
Source: Orphanet J Rare Dis. 2021 Jun 10;16:266. doi: 10.1186/s13023-021-01910-5 (PMC8191011; doi:10.1186/s13023-021-01910-5)
Supplement: Supplementary file 1 — Additional file 1. Patient and caregiver surveys. [file 13023_2021_1910_MOESM1_ESM.docx]

A. Socio-demographic data

You are:


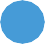


1

- **Patient with ATTRv**
- **ATTRv gene mutation carrier**
- **Family member of a patient with ATTRv**

Do you have an ATTRv mutation with polyneuropathy?


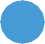


2

- **Yes**
- **No**

What is the FAP stage of your disease?


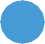


3

- **Carrier without symptoms**
- **Stage I: Does not require support or cane**
- **Stage II: Walking difficulties, does require support or cane**
- **Stage III: patient confined to a bed or wheelchair**

How long has it been since you were diagnosed with ATTRv amyloidosis?


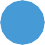


4

- **Not applicable**
- **Less than 2 years**
- **2 - 5 years**
- **5 -10 years**
- **More than 10 years**

Indicate your gender


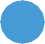


5

- **Female**
- **Male**
- **Prefer not to say**

Indicate your age


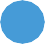


6

- **18 - 30 years old**
- **30 - 40 years old**
- **40 - 50 years old**
- **More than 50 years old**

Indicate your marital status


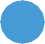


7

- **Single**
- **Married or in a civil partnership**
- **Separated**
- **Divorced**
- **Widowed**

How many children do you have?


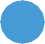


8

- **0**
- **1**
- **2**
- **3**
- **More than 3**

What is your education level?


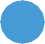


9

- **Without studies**
- **Primary or secondary education**
- **Professional training**
- **University education or higher**

What is your employment status?


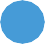


10

- **Employed**
- **Unemployed**
- **On leave from work**
- **Retired**
- **Student**

B. Family history of ATTRv

Did any of your parents become ill from ATTRv?


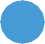


11

- **No**
- **Mother**
- **Father**
- **Both**

Are any of your parents deceased due to ATTRv?


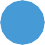


12

- **No**
- **Mother**
- **Father**
- **Both**

(if applicable) How old was your mother/father when she/he became ill with ATTRv?


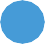


13

- **18 - 30 years old**
- **30 - 40 years old**
- **More than 40 years old**

(if applicable) How old was your mother/father when she/he died of ATTRv?


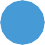


14

- **18 - 30 years old**
- **30 - 40 years old**
- **40 - 50 years old**
- **More than 50 years old**

Apart from your parents, do you have a family member diagnosed with ATTRv??


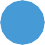


15

- **0**
- **1**
- **2**
- **More than 2**

(if applicable) How old were you when your mother/father became sick with ATTRv?


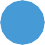


16

- **Less than18 years old**
- **18 - 25 years old**
- **More than 25 years old**

(if applicable) How old were you when your mother/father died from ATTRv?


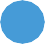


17

- **Less than18 years old**
- **20 - 25 years old**
- **More than 25 years old**

Have you ever cared for a family member suffering from ATTRv?


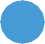


18

- **No**
- **Mother**
- **Father**
- **Another family member**

C. Influence, knowledge of /contact with ATTRv on own diagnosis

Were there any factors that helped you understand your own ATTRv diagnosis?


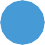


19

- **None**
- **The illness of my parents**
- **The illness of another family member**
- **Other (specify details):**

What is your main source of information about the disease?


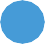


20

- **Patients' Association**
- **My doctor**
- **Relatives with the disease**
- **The Internet**
- **Other (specify details):**

D. Diagnosis, genetic counselling, and time to results

Did you have access to genetic counselling for ATTRv?


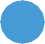


21

- **Yes**
- **No**

(if applicable) What motivated your request for TTR genetic counselling?


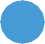


22

- **The illness of my parents**
- **The illness of my relatives**
- **My doctor’s request**
- **My own request**

Did you have difficulty accessing genetic testing?


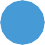


23

- **Yes**
- **No**

(if applicable) What barriers were did you encounter in accessing genetic testing?


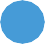


24

Were you afraid of finding out the results of the genetic tests?


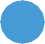


25

- **Yes**
- **No**

How long did it take for you to receive the results of the genetic tests from the time you took them?


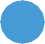


26

- **Less than 6 months**
- **6 months- 1 year**
- **More than 1 year**

E. Impact of ATTRv on the patient's life

Has your ATTRv diagnosis influenced your work/study plans?


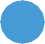


27

- Yes
- No

If so, please specify how:


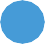


28

Has your ATTRv diagnosis influenced your plans to have your own family?


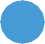


29

- **Yes**
- **No**

If so, please specify how:


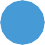


30

Have you been forced to move because of your ATTRv?


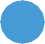


31

- **Yes**
- **No**

Have you needed to hire a caregiver because of your illness?


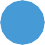


32

- **Yes**
- **No**

Has the disease influenced your income?


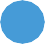


33

- Yes
- No

Has the disease affected your relationships with friends?


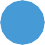


34

- Yes
- No

Do you have any state support because of your illness?


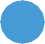


35

- Yes
- No

If so, please specify which:


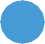


36

Do you belong to an association related to your disease?


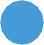


37

- Yes
- No

If so, please specify which:


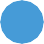


38

F. Psychological or emotional impact after ATTRv diagnosis

Have you had any psychological/psychiatric issues associated with your ATTRv diagnosis?


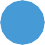


39

- Yes
- No

If so, which one(s)?


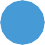


40

- Depression
- Anxiety
- Other (specify):

(if it is applicable) When have these problems occurred?


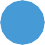


41

- At the time of the diagnosis
- The year following the diagnosis
- Several years after the diagnosis
- These are permanent problems from the onset of the first symptoms.

(if applicable) Have you required any professional help you to deal with these issues?


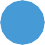


42

- Yes
- No

If so, what type of professional has helped you?


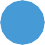


43

- Psychologist
- Psychiatry
- Ohter (specify):

(if applicable) Have you required any medication to treat these problems?


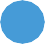


44

- Yes
- No

If so, what type of medication?


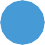


45

G. Areas for improvement

Please rate from 1 to 10 your satisfaction with the following aspects (1= not at all satisfied, 10= very satisfied).

Care received in relation to your diagnosis


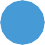


46


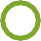


1


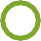


2


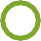


3


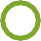


4


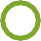


5


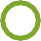


6


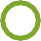


7


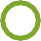


8


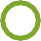


9


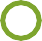


10

Care received in relation to your illness


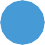


47


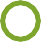


1


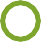


2


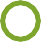


3


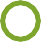


4


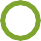


5


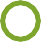


6


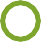


7


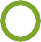


8


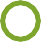


9


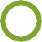


10

What do you think could be improved in the care you received in terms of your diagnosis?


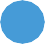


48

- Medical care
- Psychological care
- Social aspects
- Other (specify):

If so, what kind of improvements in ATTRv diagnosis do you think are needed?


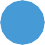


49


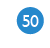


What do you think could be improved in the care you received in terms of your disease?

- Medical care
- Psychological care
- Social aspects
- Other (specify):


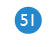


If so, what kind of improvements in ATTRv diagnosis do you think are needed?

***Bibliography***

**-** Lopes A, Sousa A, Fonseca I, et al. Life paths of patients with transthyretin-related familial amyloid polyneuropathy Val30Met: a descriptive study. J Community Genet. 2018 Jan;9(1):93-99. doi: 10.1007/s12687-017-0338-0.

A. Socio-demographic data

What is your relationship with the patient diagnosed with ATTRv amyloidosis for whom you care?


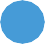


1

- Parents
- Son/daughter
- Spouse
- Relative (specify):

How long has it been since the patient was diagnosed with ATTRv amyloidosis?


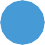


2

- Less than 2 years
- 2 - 5 years
- 5 -10 years
- More than10 years


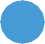


3

What is the PAF stage of the disease?

- Carrier without symptoms
- Stage I: Does not require support or cane
- Stage II: Walking difficulties, does require support or cane
- Stage III: patient confined to a bed or wheelchair

How many hours a day do you spend caring for this patient?


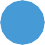


4

- Less than 2
- 2 - 5 hours
- 5 - 10 hours
- More than 10 hours

Indicate you gender


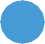


5

- **Female**
- **Male**
- **Prefer not to say**

Indicate your age


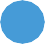


6

**
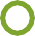
 18 - 30 years old**

**
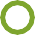
 30 - 40 years old**

**
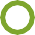
 40 - 50 years old**

**
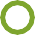
 More than 50 years old**

Indicate your marital status


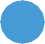


7

- **Single**
- **Married or in a civil partnership**
- **Separated**
- **Divorced**
- **Widowed**

How many children do you have?


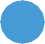


8

- **0**
- **1**
- **2**
- **3**
- **More than 3**

What is your education level?


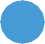


9

- **Without studies**
- **Primary or secondary education**
- **Professional training**
- **University education or higher**

What is your employment status?


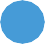


10

- **Employed**
- **Unemployed**
- **On leave from work**
- **Retired**
- **Student**

Do you suffer from any relevant illness?


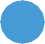


11

- **Yes**
- **No**

If so, which disease?


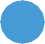


12

B. Patient symptoms

Please indicate only those ATTRv symptoms that the patient for whom you are caring has experienced and rate on a scale of 1 to 10 the burden this symptom has placed on you as caregiver in the last year (1= not at all problematic, 10= very problematic).

Difficulty in walking, muscle weakness


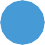


13


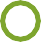


1


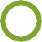


2


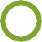


3


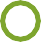


4


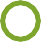


5


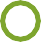


6


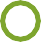


7


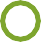


8


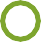


9


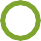


10

Numbness, cramps, swelling or pain in the legs or feet


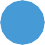


14


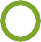


1

2

3

4

5

6

7

8

9

10

Tiredness, dizziness, loss of balance

15

1

2

3

4

5

6

7

8

9

10

Constipation, diarrhoea, nausea/vomiting, weight loss/loss of appetite

16

1

2

3

4

5

6

7

8

9

10

Faecal or urinary incontinence

17

1

2

3

4

5

6

7

8

9

10

Visual disturbances

18

1

2

3

4

5

6

7

8

9

10

Erectile dysfunction

19

1

2

3

4

5

6

7

8

9

10

Anxiety/depression

20

1

2

3

4

5

6

7

8

9

10

Insomnia

21

1

2

3

4

5

6

7

8

9

10

Respiratory distress, tachycardia

22

1

2

3

4

5

6

7

8

9

10

Carpal tunnel syndrome, loss of manual dexterity

23

1

2

3

4

5

6

7

8

9

10

Other:

24

C.Treatments the patient has had recourse to

Indicate only those treatments that the patient for whom you are caring has had recourse to and the degree of satisfaction that the patient has obtained from them in the last year (1= not at all satisfied, 10= very satisfied).

Treatment of tiredness

25

1

2

3

4

5

6

7

8

9

10

Treatment of digestive symptoms

26

1

2

3

4

5

6

7

8

9

10

Neuropathic pain treatment

27

1

2

3

4

5

6

7

8

9

10

Anxiety/depression treatment

28

1

2

3

4

5

6

7

8

9

10

Insomnia treatment

29

1

2

3

4

5

6

7

8

9

10

Treatment of cardiac function (e.g. diuretics)

30

1

2

3

4

5

6

7

8

9

10

Blood pressure treatment

31

1

2

3

4

5

6

7

8

9

10

Treatment of visual disturbances

32

1

2

3

4

5

6

7

8

9

10

D. Impact of ATTRv on the life of the caregiver

Please rate on a scale of 1 to 10 the burden of caring for a patient with ATTRv in the last year in the aspects indicated (1= not at all problematic, 10= very problematic).

Your physical health

33

1

2

3

4

5

6

7

8

9

10

If so, please specify how

34

Have you received medication for this reason? Which one?

35

Your emotional well-being

36

1

2

3

4

5

6

7

8

9

10

If so, please specify how:

37

Have you received medication for this reason? Which one?

38

Your study /work plans

39

1

2

3

4

5

6

7

8

9

10

If yes, please specify how:

40

Your family/leisure plans

41

1

2

3

4

5

6

7

8

9

10

If so, please specify how:

42

Your income

43

1

2

3

4

5

6

7

8

9

10

Do you have any kind of state support due to caregiving?

44

- **Yes**
- **No**

If so, which one?

45

Do you belong to an ATTRv-related association?

46

**Yes No**

If so, which one?

47

***Bibliography***

- Lopes A, Sousa A, Fonseca I, et al. Life paths of patients with transthyretin-related familial amyloid polyneuropathy Val30Met: a descriptive study. J Community Genet. 2018 Jan;9(1):93-99. doi: 10.1007/s12687-017-0338-0.
